# Identifying functional dysregulation of *NOD2* variant Q902K in patients with Yao syndrome

**DOI:** 10.1186/s13075-024-03286-w

**Published:** 2024-02-23

**Authors:** Jingyuan Zhang, Yi Luo, Bingxuan Wu, Xin Huang, Mengzhu Zhao, Na Wu, Junke Miao, Ji Li, Lei Zhu, Di Wu, Min Shen

**Affiliations:** 1 Department of Rare Diseases, Peking Union Medical College Hospital (PUMCH), Chinese Academy of Medical Sciences & Peking Union Medical College; State Key Laboratory of Complex Severe and Rare Diseases, PUMCH; Department of Rheumatology and Clinical Immunology, PUMCH; National Clinical Research Center for Dermatologic and Immunologic Diseases (NCRC-DID), Ministry of Science & Technology; Key Laboratory of Rheumatology and Clinical Immunology, Ministry of Education, Beijing, 100730 China; 2 Department of Gastroenterology, Peking Union Medical College Hospital (PUMCH), Chinese Academy of Medical Sciences & Peking Union Medical College, Beijing, 100730 China; 3 Department of Pharmacology, Institute of Basic Medical Sciences, Chinese Academy of Medical Sciences and School of Basic Medicine, Peking Union Medical College, Beijing, 100005 China; 4 Department of Rheumatology and Clinical Immunology, Peking Union Medical College Hospital (PUMCH), Chinese Academy of Medical Sciences & Peking Union Medical College; National Clinical Research Center for Dermatologic and Immunologic Diseases (NCRC-DID), Ministry of Science & Technology; State Key Laboratory of Complex Severe and Rare Diseases; Key Laboratory of Rheumatology and Clinical Immunology, Ministry of Education, Beijing, 100730 China

**Keywords:** Yao syndrome, Nucleotide-binding oligomerization domain containing 2 (*NOD2*), Pathogenesis, Systemic autoinflammatory diseases

## Abstract

**Background and objectives:**

The study investigated the pathogenesis of Yao syndrome (YAOS), a rare systemic autoinflammatory disease associated with the nucleotide-binding oligomerization domain containing 2 (*NOD2*) gene variants.

**Methods:**

RNA sequencing analyses were used to detect transcriptomic profile changes. Immunoblot and immunohistochemistry were used to examine the NOD2-mediated inflammatory signaling pathways and ELISA was used to detect cytokines.

**Results:**

Transcriptome analysis of YAOS revealed NOD-like receptor signaling pathway enrichment. Compared with HCs, P-RIP2, p-p65, p-p38, p-ERK, and p-JNK notably increased in PBMCs of a patient with YAOS. P-RIP2, p-p65, and p-p38 elevated in small intestinal mucosa tissues. P-p65 and p-p38 in synovial tissues from YAOS were higher than those in patients with rheumatoid arthritis (RA) and osteoarthritis (OA). Serum interleukin (IL)-6 level along with tumor necrosis factor (TNF)-α and IL-6 secreted from PBMCs were markedly higher in patients with YAOS in comparison to healthy controls (HCs). The supernatants of synovial cells from a patient with YAOS showed substantially higher IL-1β and IL-6 levels than those of RA and OA. Canakinumab therapy of a Q902K heterozygous patient with YAOS resulted in notable clinical improvement.

**Conclusion:**

Overproduction of pro-inflammatory cytokines and the hyperactivation of NOD2-mediated signaling pathways were found in the *NOD2* variant Q902K patient with YAOS. NOD2-RIP2-MAPK pathway might play a pivotal role in the pathogenesis of YAOS. These results provide new perspectives for targeted therapies in YAOS.

**Supplementary Information:**

The online version contains supplementary material available at 10.1186/s13075-024-03286-w.

## Key messages

Abnormal NOD2-RIP2-MAPK pathways were activated in YAOS with *NOD2* variant Q902K.

**Supplementary Information:**

The online version contains supplementary material available at 10.1186/s13075-024-03286-w.

## Introduction

Yao syndrome (YAOS, OMIM# 617321), originally named nucleotide-binding oligomerization domain containing 2 (*NOD2*)-associated autoinflammatory disease (NAID), is a systemic autoinflammatory disease (SAID) first reported by Yao and colleagues in 2011 [1]. Patients with YAOS are mostly white adults with a female predominance and exhibit periodic fever, dermatitis, arthralgia/arthritis, serositis, sicca-like symptoms, and non-specific gastrointestinal symptoms, as well as distal lower extremity swelling [2]. Recently, different genotypes and clinical phenotypes have been found in Chinese patients with YAOS [3]. The complex clinical manifestations of this disorder and a lack of awareness often result in delayed diagnosis. Devoid of the most efficacious drugs, treatments for YAOS remain empirical with glucocorticoids and sulfasalazine as the first-line choices; therefore, further investigations on the pathogenesis of YAOS are warranted for target therapies [4, 5].

*NOD2* variants have been linked to YAOS, Blau syndrome (BS), and Crohn’s disease (CD) [2, 6]. The *NOD2* gene is located in chromosome 16q12-21. NOD2 protein is composed of N-terminal caspase recruitment domains (CARDs), central nucleotide binding and oligomerization domain (NBD), and C-terminal leucine-rich repeats (LRRs) [7]. NOD2 recognizes and combines with pathogen-associated molecular patterns (PAMPs), then receptor interaction protein-2 (RIP2) and nuclear factor kappa-B (NF-κB)/mitogen-activated protein kinases (MAPKs) inflammatory signaling are activated, which leads to the overproduction of interleukin (IL)-1β, IL-6, tumor necrosis factor (TNF)-α, and antimicrobial peptides [8–10].

Unfortunately, the exact role of NOD2 in the pathogenesis of YAOS remains unknown. Previous studies found that *NOD2* variants of patients with YAOS were including IVS8^+158^, IVS8^+158^/R702W, IVS8^+158^/1007 fs, IVS8^+158^/G908R, IVS8^+158^/V955I, and some rare variants [2, 11, 12]. Either IVS8^+158^ or IVS8^+158^/R702W variants altered *NOD2* transcript levels instead of transcript splicing. Patients with only IVS8^+158^ had enhanced NOD2 expression, basal p38 MAPKs activity, and elevated IL-6 which were further increased after muramyl dipeptide (MDP) stimulation, while haplotype IVS8^+158^/R702W patients had reduced NOD2 expression along with decreased NF-κB pathway activation and MDP-induced TNF-α production [13].

In contrast to white adults with YAOS, our team reported that Chinese Han patients with YAOS predominantly had novel *NOD2* (NM_022162.3) variants in exon 7 (Q902K) and exon 4 (R541P, Y514H) [3]. However, the functional activity of these novel *NOD2* variants underlying YAOS has not yet been investigated. In this study, we examined NOD2 expression, RIP2, and NF-κB/MAPK inflammatory pathways activation, and proinflammatory cytokines in serum, cells, and tissues from Chinese patients with YAOS. We identified aberrant activation of NOD2-RIP2-MAPK inflammatory pathways in YAOS. These results may shed light on the potential mechanisms of YAOS and illuminate more immunotherapy targets.

## Methods

### Study participants

According to the diagnostic criteria of YAOS [2, 4, 14], three patients with YAOS were enrolled and followed up in our tertiary medical center from 2016 to 2022. Complete medical records were collected. Whole-exome sequencing (WES) by next-generation sequencing was conducted at the Beijing Joy Orient Translational Medicine Research Center Co., Ltd. Serum was collected from patient 1 and patient 2. Meanwhile, peripheral blood mononuclear cells (PBMCs), articular synovial tissues, and small intestinal mucosa tissues were collected from patient 1. Healthy controls (HCs) were volunteers recruited from the same center. Disease controls were three patients with CD who underwent small intestinal mucosal biopsy, three patients with rheumatoid arthritis (RA), and three patients with osteoarthritis (OA) who received joint replacement. The study was approved by the Institutional Review Board of Peking Union Medical College Hospital and written informed consents were obtained from all the participants according to the Declaration of Helsinki.

### Cells extraction, isolation, and culture

Peripheral venous blood samples were collected from patients with YAOS and HCs. PBMCs were first extracted using the Ficoll density gradient centrifugation. The cells from synovial tissues were collected from patient 1, three with RA, and three with OA.

### In vitro stimulation

PBMCs extracted from patient 1 and HCs were separately treated with 10 ng/ml lipopolysaccharide (LPS) (Sigma, L4391) and 10 μg/ml muramyl dipeptide (MDP) (Sigma, A9519). The PBMCs supernatants were collected, and proteins were extracted after 22 h.

### Enzyme-linked immunosorbent assay (ELISA)

The serum was collected from patient 1, patient 2, and HCs. Cytokine levels of IL-1β, IL-6, and TNF-α in the serum and the supernatant of PBMCs and synovial cells were detected according to the instructions of ELISA kits (ExCell Biotech, EH001, EH004, EH009).

### Western blot

Proteins were separated using polyacrylamide gel electrophoresis (SDS–PAGE), and the transfer to the PVDF membrane was undergone before being blocked in Tris-buffered-saline-Tween 20 (TBS-T) with 5% skim milk. The target protein band cut from the PVDF membrane was incubated with diluted primary antibody buffer overnight at 4 °C as follows: NOD2 antibody (Abcam ab31488, Santa Cruz sc-56168), RIP2 rabbit mAb (Cell Signaling Technology,4142), phospho-RIP2 rabbit mAb (Cell Signaling Technology,14397), NF-κB p65 mouse mAb (Cell Signaling Technology,6956), phospho-NF-κB p65 rabbit mAb (Cell Signaling Technology,3033), p38 MAPK rabbit mAb (Cell Signaling Technology,8690), phospho-p38 MAPK rabbit mAb (Cell Signaling Technology,45224511), ERK rabbit mAb (Cell Signaling Technology, 4695), phospho-ERK rabbit mAb (Cell Signaling Technology, 4370), JNK Antibody (Cell Signaling Technology, 9252), phospho-JNK rabbit mAb (Cell Signaling Technology, 4668), mouse Anti-β actin mAb (Beijing Zhong Shan-Golden Bridge Biological Technology Co., Ltd., TA-09). After rinsing with TBS-T, the membrane was probed with goat anti-rabbit IgG (H&L)-HRP conjugated antibody (Beijing Zhong Shan-Golden Bridge Biological Technology Co., Ltd., ZB-2301) and goat anti-mouse IgG (H&L)-HRP conjugated antibody (Beijing Zhong Shan-Golden Bridge Biological Technology Co., Ltd., ZB-2305) at room temperature for 1 h. ECL substrate (Thermo, 34,095) was added to develop the image.

### Immunohistochemistry staining

The small intestinal mucosa tissues and synovial tissues from patient 1 or HCs were paraffin-embedded. Immunohistochemistry staining was performed according to the instructions of PV-9000 two-step immunohistochemistry method (Beijing Zhong Shan-Golden Bridge Biological Technology Co., Ltd.). Immunohistochemistry images were observed and analyzed. Positive Pixel Count was quantified as the fraction of positive to total stained pixels.

### RNA sequencing

Total RNA was extracted from PBMCs of patient 1 and HCs using TRIzol reagent following the manufacturer’s procedure. Briefly, the total RNA quantity and purity were detected by Bioanalyzer 2100 (Agilent, CA, USA) and RNA 6000 Nano LabChip Kit (Agilent, CA, USA, 5067–1511) to ensure RIN number > 7.0. The mRNA was purified from total RNA (5 μg) using Dynabeads Oligo (dT) (Thermo Fisher, CA, USA) and fragmented into short fragments by Magnesium RNA Fragmentation Module (NEB, cat.e6150, USA). The cleaved RNA fragments were then reverse-transcribed into cDNA using SuperScript™ II Reverse Transcriptase (Invitrogen, cat.1896649, USA), which were next used to synthesize U-labeled second-stranded DNAs with *E. coli* DNA polymerase I (NEB, cat.m0209, USA), RNase H (NEB, cat.m0297, USA) and dUTP Solution (Thermo Fisher, cat.R0133, USA). An A-base was added to the blunt ends of each strand in preparation for ligation to the indexed adapters and each adapter contained a T-base overhang for ligating the adapter to the A-tailed fragmented DNA. Dual-index adapters were then ligated to the fragments, followed by size selection using AMPureXP beads. After the heat-labile UDG enzyme (NEB, cat.m0280, USA) treatment of the U-labeled second-stranded DNAs, the ligated products were then amplified using polymerase chain reaction (PCR) to form the final cDNA libraries with an average insert size for 300 ± 50 bp. Finally, 2 × 150 bp paired-end sequencing (PE150) was performed on Illumina Novaseq™ 6000 platform at the LC-Bio Technology CO., Ltd (Hangzhou, China) following the manufacturer’s recommended protocol. Reads of all samples were aligned to the reference genome with HISAT2 (https://daehwankimlab.github.io/hisat2/, version:hisat2-2.2.1) package. Differential gene expression analysis was performed by DESeq2/edgeR (version 1.22.2/3.22.5). The differentially expressed genes were selected with |log2 fold change (FC)|≥ 1 and *q* (adjusted *p*-value) < 0.05. OmicStudio tools at https://www.omicstudio.cn were utilized to perform heatmap plots, the volcano plot, and enrichment analysis of Kyoto Encyclopedia of Genes and Genomes (KEGG) pathways. In our KEGG analysis, we included pathways with *q* values < 0.1 to further investigate disease-related mechanisms. Gene set enrichment analysis (GSEA) was performed using software GSEA (http://www.gsea-msigdb.org/gsea/index.jsp, v4.1.0) and MSigDB; |normalized enrichment score (NES)|> 1, nominal *p*-val < 0.05, and false discovery rate (FDR) *q*-val < 0.25 were considered to be different in two groups [15, 16].

### NF-κB luciferase reporter assay

HEK293T (1 × 10^5^) cells were transfected with 100 ng wild-type (WT) *NOD2* plasmids (Genebio, Shanghai, China), *NOD2* Q902K (Genebio, Shanghai, China), or empty vector (EV) (Genebio, Shanghai, China), together with 100 ng Firefly NF-κB reporter plasmid (pNF-κB-Luc; Genebio, Shanghai, China) and 5 ng *Renilla* luciferase control vector (pRL-TK; Genebio, Shanghai, China). NF-κB activity was measured in the cells treated with or without 10 μg/ml muramyl dipeptide (MDP, Sigma) for 24 h using Dual-Luciferase Reporter Assay System (E1910; Promega) based on the protocol provided by the manufacturer. We measured the activities of Firefly and *Renilla* luciferase and calculated the ratio of Firefly/*Renilla* luciferase activity. Firefly/*Renilla* luciferase with and without MDP stimulation was respectively normalized to the ratio measured for MDP-stimulated and unstimulated EV groups. Each experiment was performed in triplicate.

### Bioinformatics

Molecular Evolutionary Genetics Analysis version 11 (MEGA11) [17] was used to perform multiple sequence alignment of NOD2 protein across various species. The homology model of the NOD2 was based on the template from AlphaFold [18]. DynaMut [19] was used to predict the effect of *NOD2* variants on protein flexibility and interatomic interactions.

### Statistical analysis

All data were analyzed by GraphPad Prism 7.0 (GraphPad Software, USA) and SPSS (IBM SPSS Statistics for Windows, Version 25.0, Armonk, NY) presenting as mean ± standard deviation (SD). Analysis of comparison among groups was performed by unpaired *t*-test and one-way analysis of variance (ANOVA). *p* < 0.05 was considered statistically significant.

## Results

### Case description of patients with YAOS

Patient 1 was a 49-year-old Chinese Han who reported over a decade of recurrent febrile episodes, coupled with persistent left upper abdominal pain. She also experienced moderate bilateral knee arthritis and distal lower extremity swelling. Leukocytes, erythrocyte sedimentation rate (ESR), and C-reactive protein (CRP) were significantly elevated during attacks and normalized in the intervals. Antinuclear antibodies (ANAs) and antineutrophil cytoplasmic antibodies (ANCA) were all negative. Gastroscopy, computed tomography (CT), and positron emission tomography (PET)/CT showed no significant abnormal findings in the gastrointestinal tract. She denied the family history of SAIDs. WES identified a heterozygous c. 2704C > A, p.Q902K variant in exon 7 of the *NOD2* gene [3].

**Fig. 1:**
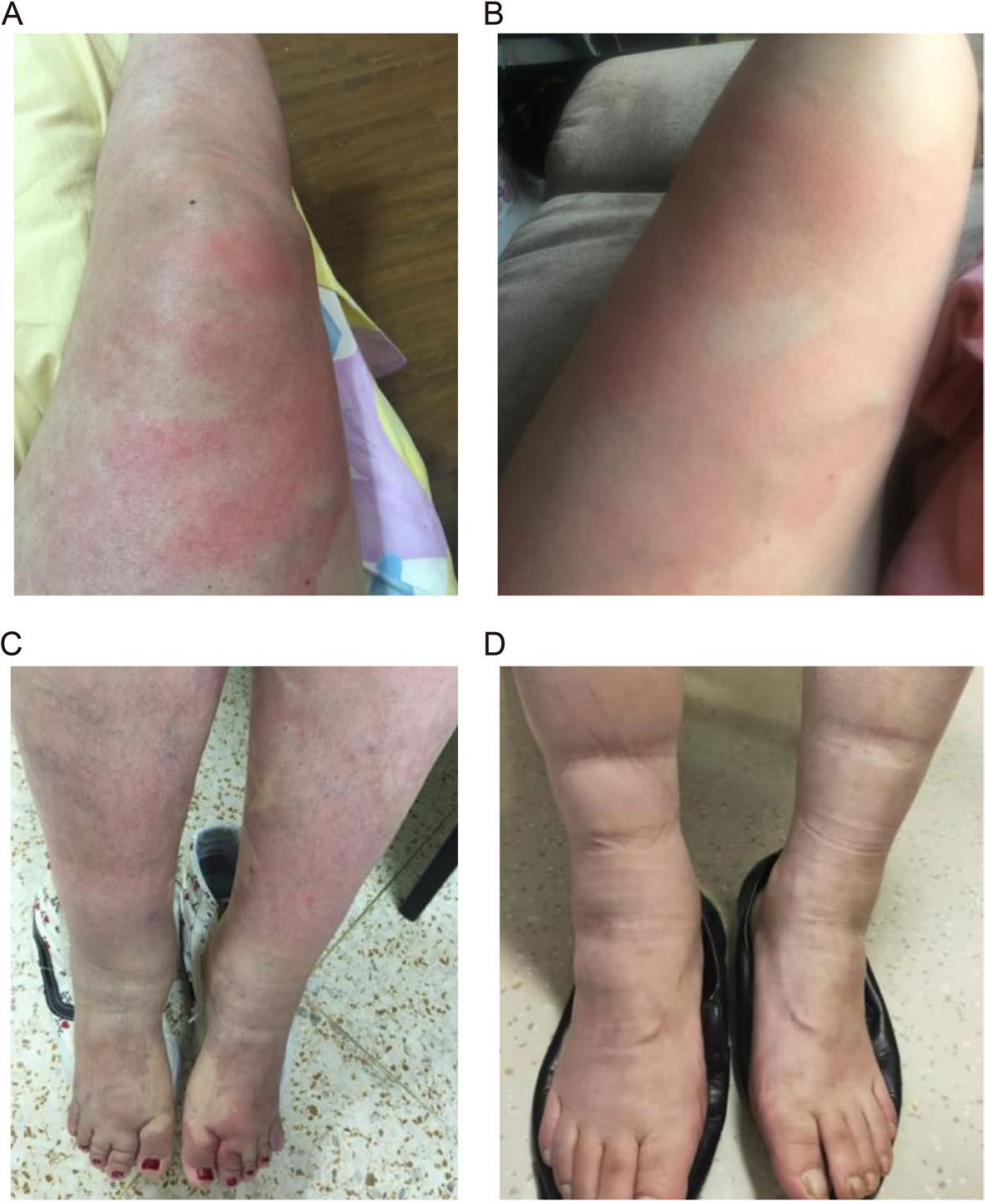
Clinical features of patients with YAOS. **A**, **B** Patchy erythema on the lower extremity of patient 2 during episodes. **C** Lower extremity swelling of patient 2. **D** Swelling in the ankles and feet of patient 3

Patient 2 was a 37-year-old female presented with a 17-year history of recurrent fever and rash. She experienced recurrent fever with a peak temperature of 40 °C, accompanied by patchy erythema on the lower extremity, lower limb joint pain, and distal lower extremity swelling (Fig. 1A–C). Symptoms lasted for about 5 days before spontaneously resolving. Initially occurring at a frequency of several times per year, the episodes have increased to several times per month in recent years, associated with nausea and vomiting, without concurrent abdominal pain, diarrhea, or chest pain. The patient denied sore throat, oral ulcers, periorbital edema, hearing loss, or lymphadenopathy. There was no family history of SAIDs. During episodes, elevated ESR and CRP levels were observed, which returned to normal during the intervals. Genetic testing identified a paternal p.Q902K variant and a maternal p.R471C variant of the *NOD2* gene.

Patient 3 was a 48-year-old male patient presented with a 3-year history of recurrent fever. The patient experienced unprovoked recurrent fever starting at the age of 45, with episodes occurring every 2 weeks or 2–3 months, lasting 3–5 days each time and reaching a peak temperature of over 39 °C. His fevers were accompanied by oral ulcers occurring more than 10 times per year, along with myalgia, lower abdominal pain, diarrhea, distal lower extremity swelling, and poor appetite (Fig. 1D). Nausea and vomiting were not present. Additionally, acne-like rash, folliculitis, and rhinophyma were observed. The patient experienced a weight loss of 10 kg and a previous episode of cerebral infarction. There were no external genital ulcers, hearing loss, uveitis, headache, or chest pain. During episodes, elevated ESR and CRP were noted, returning to normal during the intervals. He denied the family history of SAIDs. ANAs, ANCA, complement levels, immunoglobulin, and chest CT were all normal. Genetic testing identified a heterozygous *NOD2* p.Q902K variant.

Based on the typical clinical manifestations and WES results, three patients were finally diagnosed with YAOS [2, 4, 14]. The demographic and clinical features of these patients are summarized in Table 1.

**Table 1:** Demographic and clinical manifestations of patients with YAOS

Characteristics	Patient 1	Patient 2	Patient 3
Gender	Female	Female	Male
Age at diagnosis (years old)	49	37	48
Age at onset (years old)	39	20	45
Ethnicity	Han	Han	Han
Family history	No	No	No
Clinical features			
Fever	Yes	Yes	Yes
Arthritis/arthralgia	Yes	Yes	No
Gastrointestinal symptoms	Yes	Yes	Yes
Distal lower extremity swelling	Yes	Yes	Yes
Skin rash	No	Yes	Yes
Sicca-like symptoms	No	No	No
Serositis	Yes	No	No
Leukocytosis	Yes	No	No
Elevated ESR/CRP	Yes	Yes	Yes
*NOD2* variants			
Nucleotide exchanges	c.2704C > A	c.2704C > A c.1411C > T	c.2704C > A
Amino acid exchanges	p.Q902K	p.Q902K p.R471C	p.Q902K

*NOD2* Nucleotide-binding oligomerization domain containing 2, *ESR* Erythrocyte sedimentation rate, *CRP* C-reactive protein

### The 902 site of NOD2 is relatively stable during the evolutionary process

**Fig. 2:**
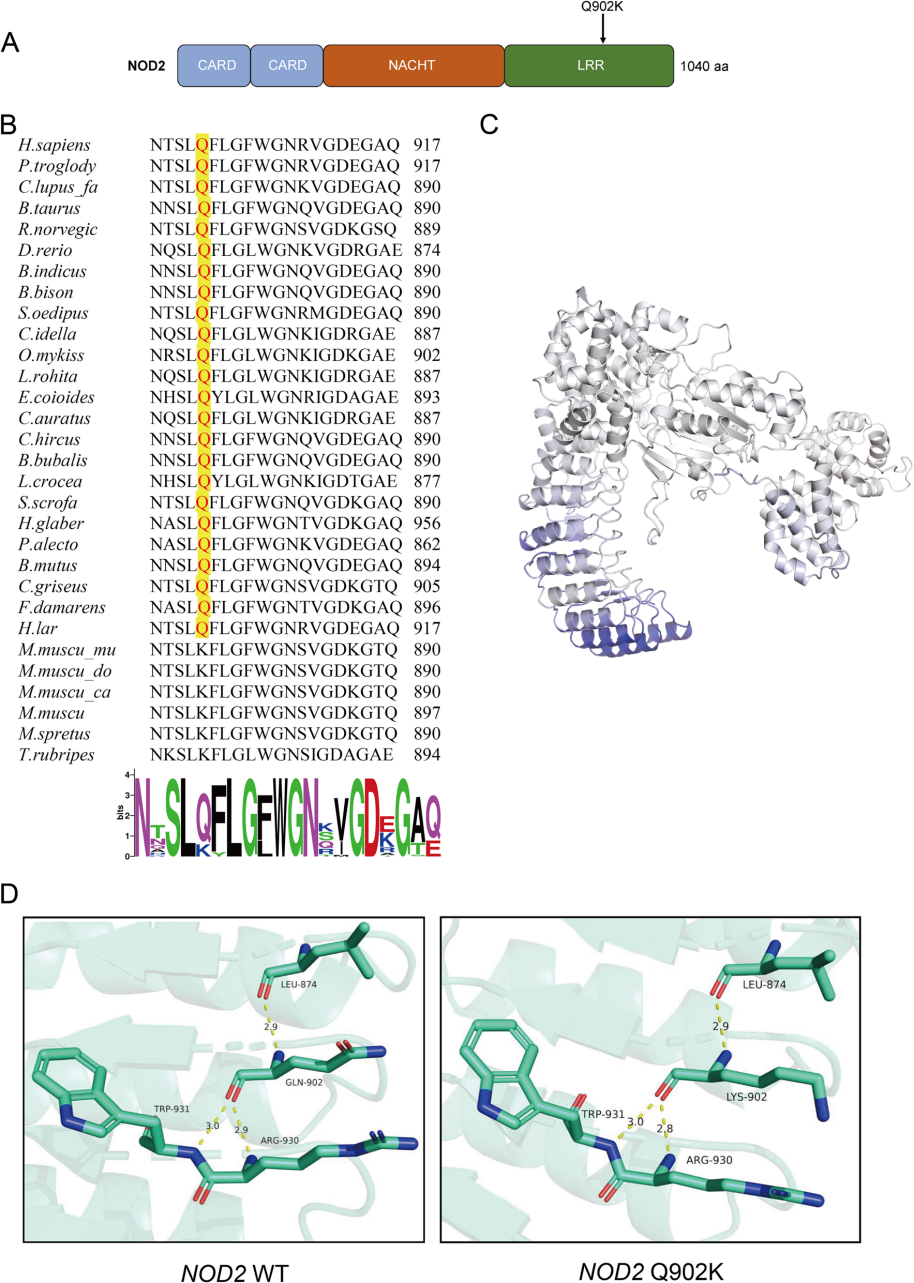
The 902 site of NOD2 is relatively stable during the evolutionary process. **A** Schematic representation of NOD2 protein structure and the location of *NOD2* variant Q902K. **B** Evolutionary conservation of the site Q902 in NOD2 across various species using MEGA11 [17] and WebLogo [21]. **C** Amino acids are colored according to the vibrational entropy change upon mutation. BLUE represented a rigidification of the structure and RED represented a gain in flexibility. Images were provided by DynaMut [19]. **D** The structural effect of *NOD2* variant Q902K was modeled based on the template from AlphaFold [18] and visualized by PyMOL Viewer software

The NOD2 protein structure and location of Q902K variant were shown (Fig. 2A). Q902 in NOD2 was conserved in 24/30 species [20] (Fig. 2B). Decreased flexibility in the LRR domain of NOD2 was predicted in Q902K variant depending on vibrational entropy energy by DynaMut [19] (Fig. 2C). Moreover, altered hydrogen bond interaction between residue 930 and amino acid 902 was visualized by PyMOL Viewer software after the substitution of lysine for glutamine at amino acid 902 (Fig. 2D). In total, these results indicated that the 902 site of NOD2 is relatively stable during the evolutionary process and *NOD2* variant Q902K may affect the protein structure.

### NOD-like receptor (NLR) signaling pathway is associated with the pathogenesis of YAOS

**Fig. 3:**
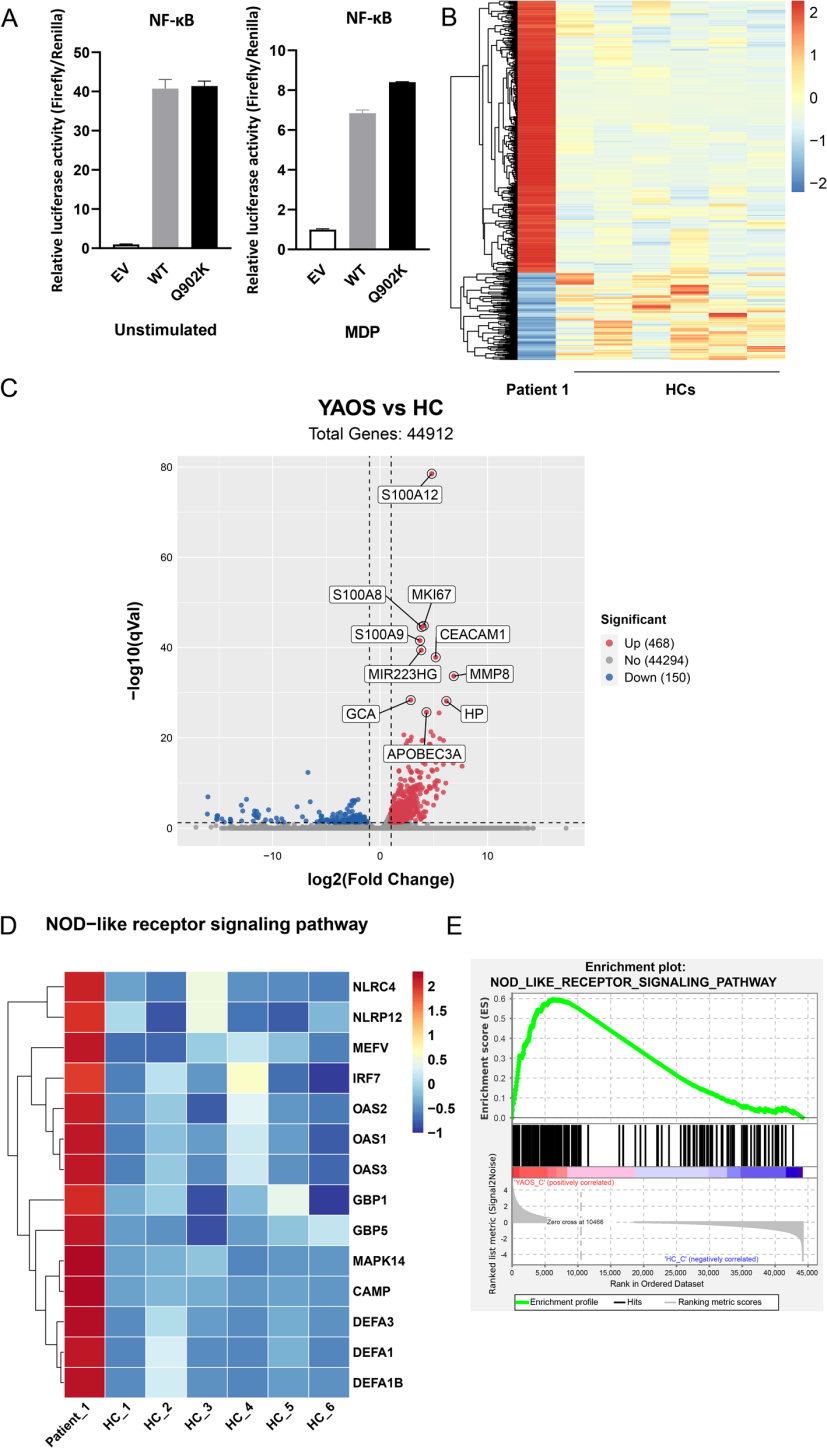
NOD-like receptor (NLR) signaling pathway is associated with the pathogenesis of YAOS. **A** NF-κB dual-luciferase reporter assay was conducted in the HEK293T cells transfected with either *NOD2*-WT or Q902K without (left) or with (right) 24 h MDP (10 μg/mL) stimulation. Fold was expressed as the Firefly (F: Lum)/*Renilla* luciferase activity (R: Lum). The values were normalized to the ratio of the EV group without (left) or with (right) MDP stimulation respectively. Analysis of samples was performed in triplicate. WT, wild type. **B**–**E** RNA sequencing of patient 1 and HC groups (*n* = 6). **B** The heatmap for DEGs comparing patient 1 and HCs. **C** Volcano plot for DEGs. **D** The heatmap of genes enriched in the NOD-like receptor signaling pathway with KEGG analysis. **E** GSEA analysis. DEGs, differentially expressed genes; KEGG, Kyoto Encyclopedia of Genes and Genomes; GSEA, gene set enrichment analysis

BS-related *NOD2* variants, which are considered gain-of-function, show increased MDP-independent NF-κB activation in vitro [22]. Intriguingly, *NOD2* Q902K-transfected cells did not demonstrate an increase in basal and MDP-stimulated NF-κB activity when compared to wild-type NOD2, which indicated that Q902K may not affect the activation of NF-κB transcription factor (Fig. 3A). To further explore the functional changes associated with Q902K, transcriptome analysis (RNA-sequencing) was then performed. The heatmap of the clustering analysis revealed significant differences in gene expressions between YAOS and HCs (Fig. 3B). There was a total of 618 differentially expressed genes, including 468 upregulated genes and 150 downregulated genes (Fig. 3C). Kyoto Encyclopedia of Genes and Genomes (KEGG) analysis (Fig. 3D, Supplementary Fig. 1A) and Gene set enrichment analysis (GSEA) revealed that enriched NLR signaling pathways were upregulated in YAOS (Fig. 3E, Supplementary Fig. 1B; Supplementary Tables 1 and 2). These results demonstrated abnormal NLR pathway activation in YAOS.

### NOD2-RIP2-MAPK inflammatory pathways were activated in YAOS

**Fig. 4:**
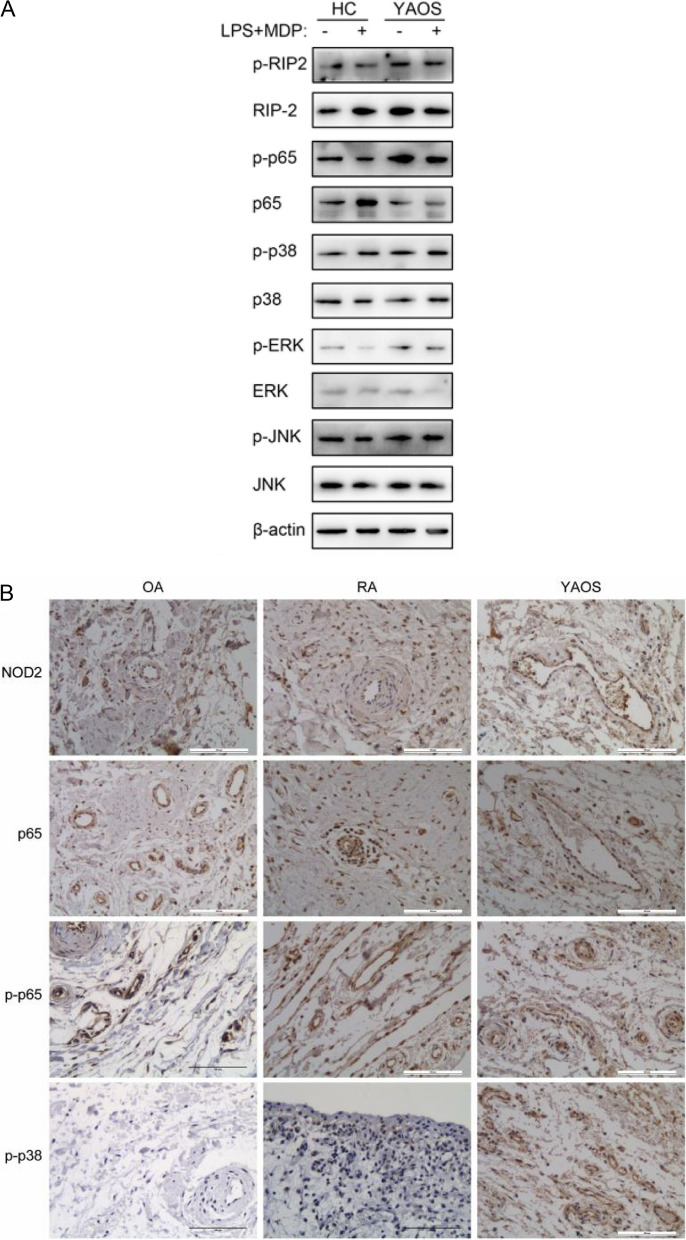
NOD2-RIP2-MAPK inflammatory pathways were activated in PBMCs and synovial tissues from YAOS. **A** Western blot analysis of NOD2 signaling pathways in PBMCs from patient 1 treated with LPS (10 ng/ml) combined with MDP (10 μg/ml) stimulation. β-actin was used as a loading control. HC (*n* = 3). Representative images of three independent experiments were shown. **B** Representative images of IHC staining for abnormal NOD2 pathway activation in synovial tissues from patient 1. OA (*n* = 3); RA (*n* = 3). HC, healthy control; OA, osteoarthritis; RA, rheumatoid arthritis; IHC, immunohistochemistry

As one of the innate immune system’s pattern recognition receptors (PRRs), NOD2 recognizes and binds with ligand MDP to initiate NOD2-associated inflammatory signal pathway which induces inflammation [8]. Combined with the previous study which indicated aberrant NOD2 function and activation in YAOS [13], we further explored the effect of *NOD2* Q902K on the activation of the NOD2 protein and the downstream inflammatory signal pathway. Immunoblot and immunohistochemistry were used to detect the pivotal expression of NLR signaling. Immunoblot of PBMCs lysates from patient 1 revealed that the basal levels of RIP2 and p-RIP2 as well as p-p65 and MAPK pathway associated p-p38, p-ERK, and p-JNK were elevated in PBMCs of patient 1 when compared with HCs. By contrast, the basal expression of p65 and p38 was reduced, and the basal expression of ERK and JNK paralleled that of HCs. Furthermore, upregulated expressions of p-RIP2, p65, p38, p-p38, p-ERK, JNK, and p-JNK were detected in YAOS after LPS combined with MDP stimulation (Fig. 4A). However, the PBMCs of neither YAOS nor HCs had a distinct band of the NOD2 protein.

**Fig. 5:**
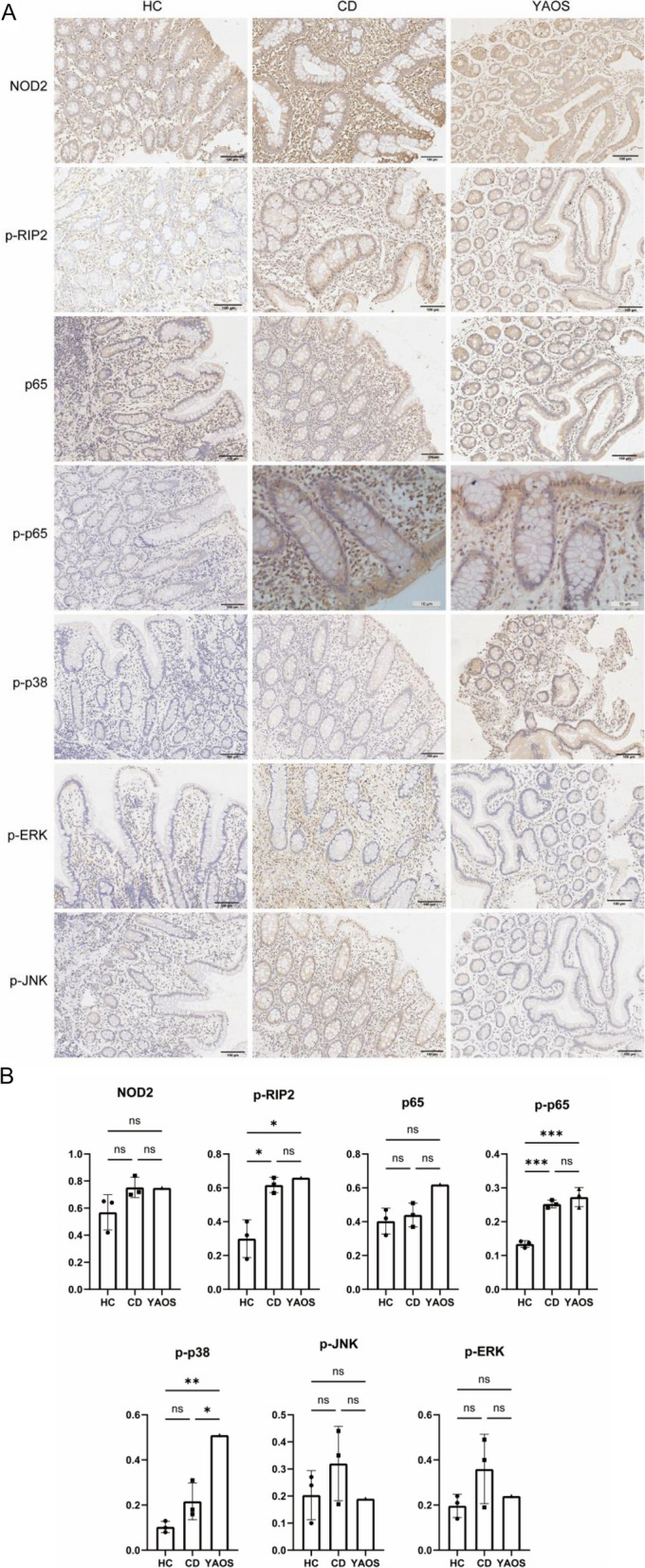
NOD2-RIP2-MAPK inflammatory pathways were activated in small intestinal mucosa tissues from YAOS. **A** Representative images of IHC staining for abnormal NOD2 pathway activation in small intestinal mucosa tissues from patient 1. **B** Quantitative results of IHC in small intestinal mucosa tissues. Positive Pixel Count was quantified as the fraction of positive to total stained pixels using Aperio ImageScope. HC (*n* = 3); CD (*n* = 3). IHC, immunohistochemistry; HC, healthy control; CD, Crohn’s disease. ns represents no statistical significance. *p < 0.05, ** *p* < 0.01, *** *p* < 0.001

We next undertook immunohistochemistry. The synovial tissues from patient 1 were positive for NOD2 and p65 in YAOS. We compared the expressions of NOD2 signaling pathways of YAOS with those of OA and RA. P-p65 in YAOS was significantly higher than that in OA. The expression of p-p38 significantly increased in YAOS compared with that of OA and RA (Fig. 4B). Immunohistochemistry of small intestinal mucosa tissue from patient 1 showed that NOD2 expression was more increased in CD and YAOS than in HCs. The expressions of p-RIP2 (*p* < 0.05) and p-p65 (*p* < 0.001) were more pronounced in CD and YAOS compared with those of HCs. Significantly elevated levels of p-p38 were found in YAOS compared with that in HCs (*p* < 0.01) and CD (*p* < 0.05). The levels of p-ERK and p-JNK in the small intestinal mucosa had no significant difference between HCs and YAOS, and CD (Fig. 5A, B).

These findings indicated that patients with YAOS had increased activation of RIP2 protein, p-p65, and MAPK inflammatory signaling.

### Pro-inflammatory cytokines IL-1β, TNF-α, and IL-6 were elevated in YAOS

**Fig. 6:**
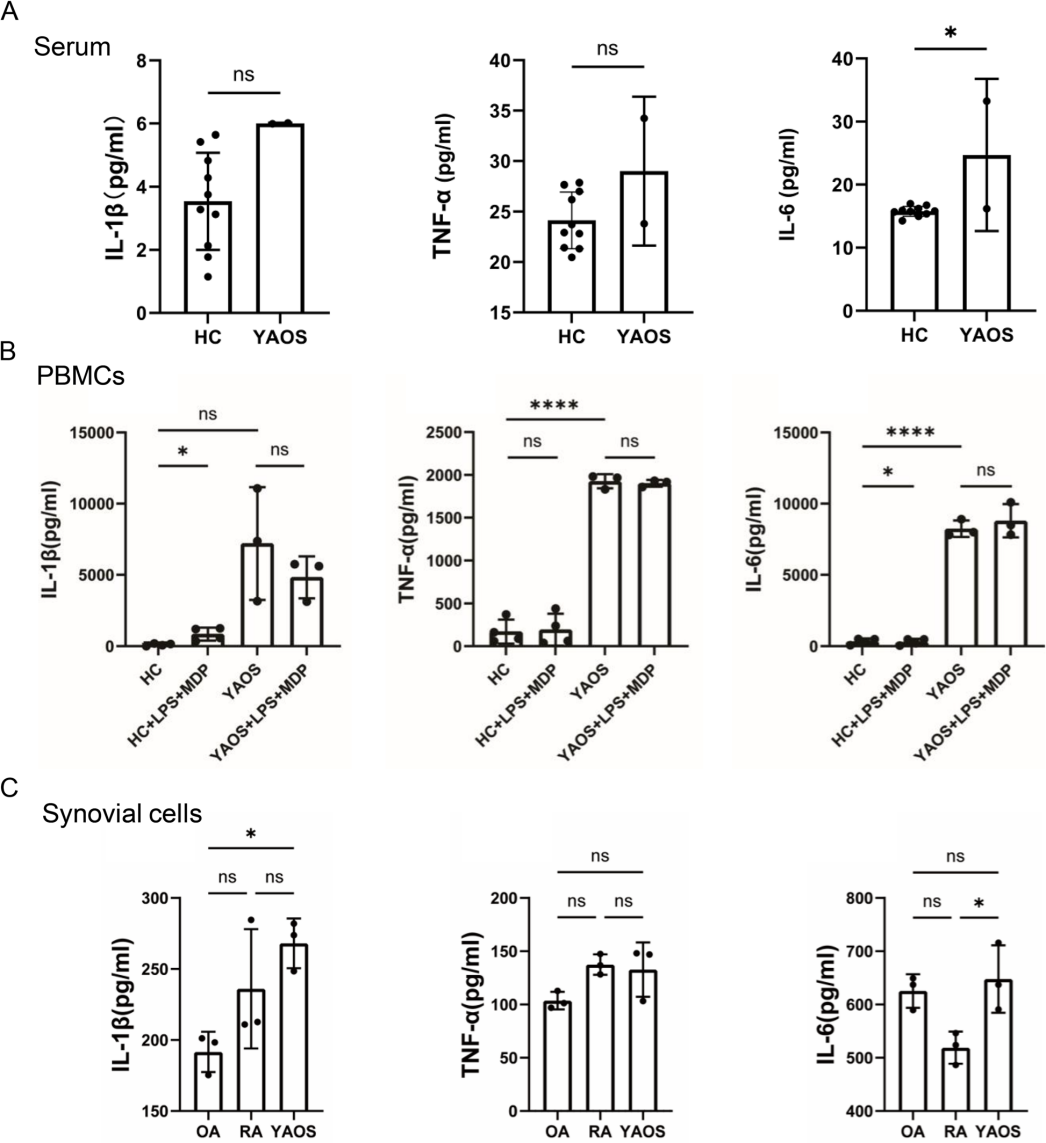
Pro-inflammatory cytokines IL-1β, TNF-α, and IL-6 were elevated in YAOS. **A** Serum IL-1β, TNF-α, and IL-6 levels. Serum was collected from patient 1 and patient 2; HC (*n* = 9). Analysis of YAOS was performed in duplicate. * *p* < 0.05, unpaired *t*-test. **B** The supernatants of PBMCs from patient 1 were collected after 22 h stimulation of LPS (10 ng/ml) and MDP (10 μg/ml). HC (*n* = 4). Analysis of YAOS was performed in triplicate. **C** Cytokines IL-1β, TNF-α, and IL-6 levels in the supernatant of synovial cells from patient 1. OA (*n* = 3); RA (*n* = 3). Analysis of YAOS was performed in triplicate. HC, healthy control; OA, osteoarthritis; RA, rheumatoid arthritis. Cytokines IL-1β, TNF-α, and IL-6 levels were detected using ELISA. mean ± SD was shown. ns represents no statistical significance. * *p* < 0.05, **** *p*-value < 0.0001, one-way ANOVA

Patients with SAIDs commonly present with inflammatory manifestations accompanied by significantly elevated pro-inflammatory cytokines [23]. To understand inflammatory patterns that exist in YAOS with Q902K variant, ELISA was used to detect cytokine profiles of patients in serum, supernatants of PBMCs, and synovial cells. We found that serum IL-6 was substantially increased in YAOS (patient 1 and patient 2) (*p* < 0.05) compared with that in HCs (Fig. 6A). Basal TNF-α and IL-6 levels in the supernatants of PBMCs from patient 1 were substantially higher than those from HCs (*p* < 0.0001). There was no significant difference regarding IL-1β, TNF-α, and IL-6 levels in PBMCs supernatants from YAOS before and after the stimulation of LPS combined with MDP. In contrast, after LPS combined with MDP stimulation, the release of IL-1β was significantly increased in HCs (*p* < 0.05) (Fig. 6B). Compared with OA, significantly increased IL-1β secretion was found in the supernatants of the articular synovial cells from patient 1 (*p* < 0.05). Moreover, the secretion of IL-6 was significantly higher in YAOS than that of RA (*p* < 0.05) (Fig. 6C). These results demonstrated that patients with YAOS had higher levels of pro-inflammatory cytokines and inflammation responses.

### IL-1 inhibitor was effective for a Q902K patient with YAOS

Patient 1 was initially treated with high doses of prednisone (40 mg) daily and sulfasalazine, and the disease remained stable. However, she presented with fever and worsening arthritis after prednisone tapering. Subsequently, the patient tried various DMARDs (methotrexate, leflunomide, mycophenolate, thalidomide, and tacrolimus) but did not respond as expected. Then, she received multiple biologics including etanercept, adalimumab, and tocilizumab as well as tofacitinib, yet still had difficulty in tapering prednisone. Finally, canakinumab was given (150 mg subcutaneously every 8 weeks), all her symptoms mentioned above were significantly improved, and inflammatory indexes (CRP and ESR) returned to normal. The patient was relieved from YAOS for the next 6 months, and prednisone was successfully tapered to 5 mg per day. These data suggest that IL-1 may have a crucial role in the pathogenesis of patients with YAOS, and IL-1 inhibitors may be an appropriate therapy for them.

## Discussion

NOD2 is mainly expressed in myeloid cells, epithelial cells, hepatocytes, and endothelial cells [24–27], known as a cytosolic bacteria sensor belonging to the NLR family. It combines with RIP2 via CARD-CARD interaction, which induces downstream pathways and synergizes with Toll-like receptors (TLRs) to eliminate bacteria and maintain homeostasis [8, 28–30]. However, dysregulation of the NOD2-RIP2 pathway could result in several inflammatory diseases including BS, CD, YAOS, cancers, and autoimmune diseases [31–34]. In the present study, we preliminarily explored the pathogenesis of patients with YAOS. We found the overproduction of pro-inflammatory cytokines and abnormal activation of NOD2-RIP2-MAPK signaling. Furthermore, treatment with canakinumab for one patient with YAOS proved to be effective. This research lays the foundation for further investigation of pathogenic mechanisms and targeted therapies for YAOS.

Circulatory cytokine profiles and NOD2 function were mainly dependent on the *NOD2* haplotype [13]. Consistently, substantially elevated pro-inflammatory cytokines production and NOD2-associated pathway activation were also found in patients with YAOS carrying *NOD2* variant Q902K. Significantly increased IL-1β, IL-6, and TNF-α were detected in serum, supernatants of PBMCs, and synovial cells from patients with YAOS. Patients with SAIDs exhibit elevated levels of pro-inflammatory cytokines, suggesting that targeted biological agents may offer potential benefits for treatment [28, 29, 35]. Likewise, IL-6 inhibitor tocilizumab was proven to significantly improve symptoms of an IVS8^+158^ only patient with YAOS [13]. Patient 1 in our study had difficulty in tapering prednisone and did not respond well to various DMARDs and biologics before canakinumab treatment. Surprisingly, canakinumab therapy notably relieved her overall symptoms and was helpful for steroid tapering. It is also reported in the literature that patients with YAOS tolerated canakinumab well with clinical improvements [4, 36, 37]. Taken together, these results suggest that IL-1 may play a crucial role in the pathogenesis of YAOS. Furthermore, the efficacy of IL-1 inhibitors warrants further investigation in a larger cohort of patients with YAOS.

The transcriptomic profile found aberrant NOD-like pathway activation in patient 1, which is consistent with the hallmark of SAIDs [32, 33]. We further demonstrated that p-RIP2, p-p65, and MAPK pathway-related p-p38 expressions were markedly increased in the PBMCs, the small intestinal mucosa tissue, and the synovial tissue from patient 1. Increased basal p38 MAPK activation was also found in the IVS8^+158^ only patient with YAOS [13]. The aforementioned results showed an increased inflammatory response in YAOS and NOD2-RIP2-MAPK signaling might be critical for the pathogenesis of YAOS. RIPK2/RIP2 is crucial in regulating NOD1/2 signaling and consequent cytokines production [38–40]. Our study provides novel insights into promising RIP2-targeted therapies for YAOS.

Three main CD-associated variants are considered to be loss-of-function due to their reduced NOD2 function and impaired basal and peptidoglycan (PGN)-induced NF-κB activity in vitro [41, 42]. They were unable to suppress inflammation by enhancing the expression of NF-κB. On the other hand, BS-related *NOD2* variants (R334Q, R334W) are considered gain-of-function abnormalities, as they exhibit increased MDP-independent and dependent NF-κB activity in vitro [32, 41]. However, several studies have shown that the BS-NOD2 variants exhibit defective MDP-mediated NF-kB activation in HEK293T cells, decreased cytokine responses in monocytes, monocyte-derived dendritic cells, macrophages from BS patients [43], as well as macrophages derived from induced pluripotential stem cells (iPS cells) bearing BS associated *NOD2* mutations [44], and macrophages from R314Q mutant mice [45]. We initially conducted dual luciferase assays to further investigate the function of *NOD2* Q902K in HEK293T cells and found that Q902K did not affect the NF-κB transcription factor. We propose that NF-κB activation may not be the central mechanism in the pathogenesis of YAOS. More in vitro cell transfection experiments and animal studies are needed to support our results.

Our study had some limitations. First, due to the rarity of the disease, we had difficulty in collecting more samples. More treatment-naive patients should be included in our cohort to fully understand the pathogenesis of the disease. Besides, the tertiary structure of mutant NOD2 protein was predicted mainly through software and the effects of gene variants can be further analyzed by macromolecular docking. Additionally, more transcript experiments and studies on transcript splicing are still needed for a better understanding of YAOS.

## Conclusions

YAOS has recently been described as a genetically transitional disease (GTD), a disease status intermediate between monogenic and polygenic disorders [14]. It mainly occurs in white adults with a female predominance [11, 46, 47]. The case series of China suggested YAOS may be a global disease with genetic heterogeneity [3]. Our results preliminarily revealed the abnormal inflammatory responses and NOD2-RIP2-MAPK activation in YAOS with a novel *NOD2* variant Q902K. Canakinumab therapy of a patient with YAOS carrying *NOD2* Q902K variant had led to a notable clinical improvement. These findings provide new perspectives for better understanding the pathogenesis of YAOS and thereby for finding potential diagnostic biomarkers and therapeutic targets.

### Supplementary Information

**Additional file 1: Supplementary Table 1.** TOP 30 pathways of GSEA Enrichment in PBMCs from patient 1. **Supplementary Table 2.** Leading edge subset of NOD-like receptor signaling pathway. 

**Additional file 2: Supplementary Figure 1.** NLR signaling pathway was activated in YAOS. (A) KEGG analysis (B) GSEA analysis of patient 1 and HC groups (*n* = 6). NLR: NOD-like receptor; KEGG, Kyoto Encyclopedia of Genes and Genomes; GSEA, gene set enrichment analysis; NES, normalized enrichment scores.

## Data Availability

The original contributions presented in the study are included in the article. Data are available on reasonable request to the corresponding author.

## Abbreviations

YAOS: Yao syndrome
NOD2: Nucleotide-binding oligomerization domain containing 2
NF-κB: Nuclear factor kappa-B
IL: Interleukin
TNF: Tumor necrosis factor
HCs: Healthy controls
NAID: Nucleotide-binding oligomerization domain containing 2-associated autoinflammatory disease
SAID: Systemic autoinflammatory disease
BS: Blau syndrome
CD: Crohn’s disease
CARDs: Caspase recruitment domains
NBD: Nucleotide binding and oligomerization domain
LRRs: Leucine-rich repeats
PAMPs: Pathogen-associated molecular patterns
RIP2: Receptor interaction protein-2
MAPKs: Mitogen-activated protein kinases
WES: Whole-exome sequencing
PBMCs: Peripheral blood mononuclear cells
RA: Rheumatoid arthritis
OA: Osteoarthritis
LPS: Lipopolysaccharides
MDP: Muramyl dipeptide
ELISA: Enzyme-linked immunosorbent assay
DEGs: Differentially expressed genes
KEGG: Kyoto Encyclopedia of Genes and Genomes
GSEA: Gene set enrichment analysis
PCR: Polymerase chain reaction
WT: Wild-type
EV: Empty vector
ESR: Erythrocyte sedimentation rate
CRP: C-reactive protein
ANAs: Antinuclear antibodies
ANCA: Antineutrophil cytoplasmic antibodies
NLR: NOD-like receptor
PGN: Peptidoglycan
