# Supplementary material for: Identifying functional dysregulation of NOD2 variant Q902K in patients with Yao syndrome
Source: Arthritis Res Ther. 2024 Feb 23;26:58. doi: 10.1186/s13075-024-03286-w (PMC10885518; doi:10.1186/s13075-024-03286-w)
Supplement: Supplementary file 2 — Additional file 2: Supplementary Figure 1. NLR signaling pathway was activated in YAOS. (A) KEGG analysis (B) GSEA analysis of patient 1 and HC groups (n = 6). NLR: NOD-like receptor; KEGG, Kyoto Encyclopedia of Genes and Genomes; GSEA, gene set enrichment analysis; NES, normalized enrichment scores. [file 13075_2024_3286_MOESM2_ESM.docx]

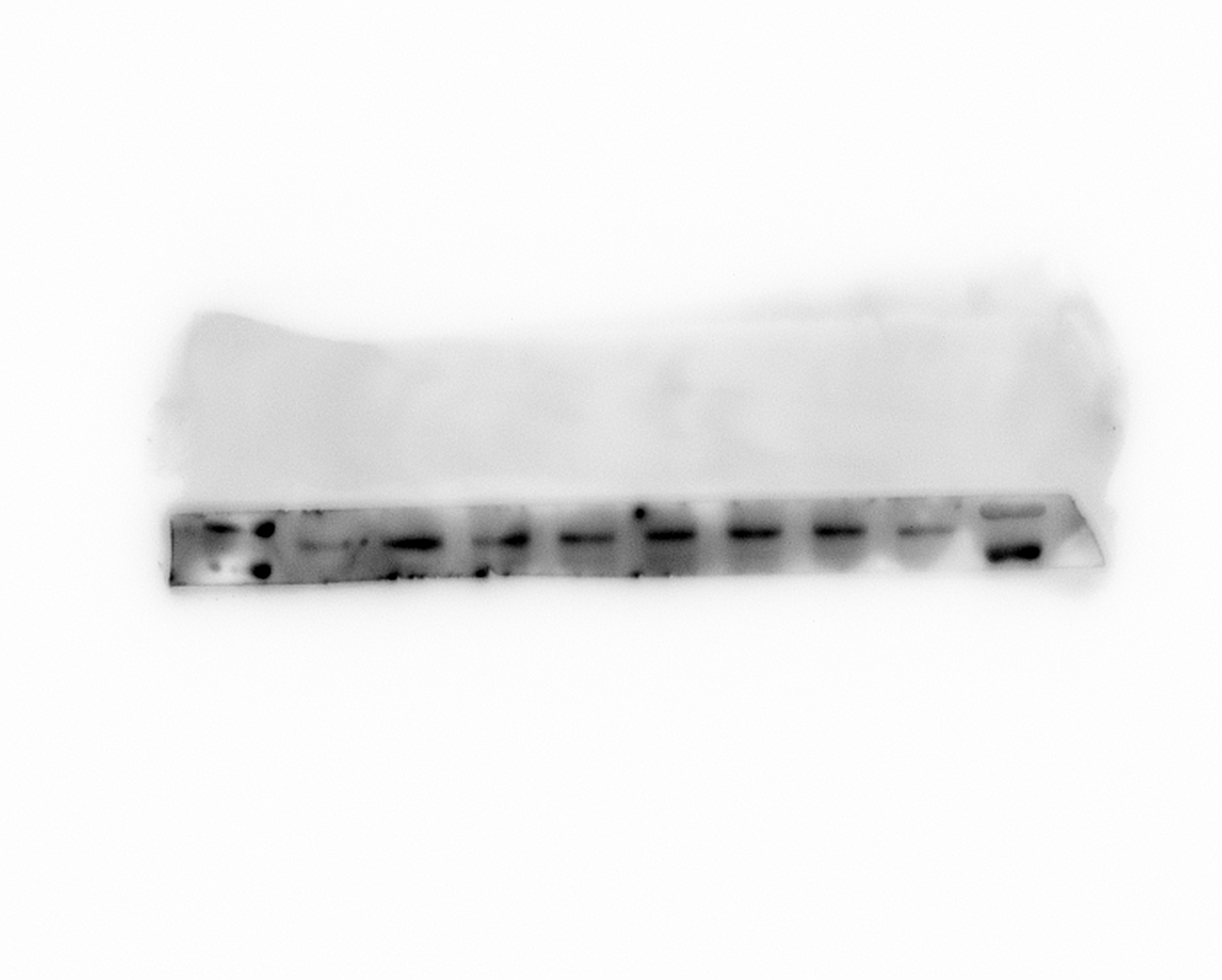


**Supplementary FIGURE 1.p-RIP2**

**
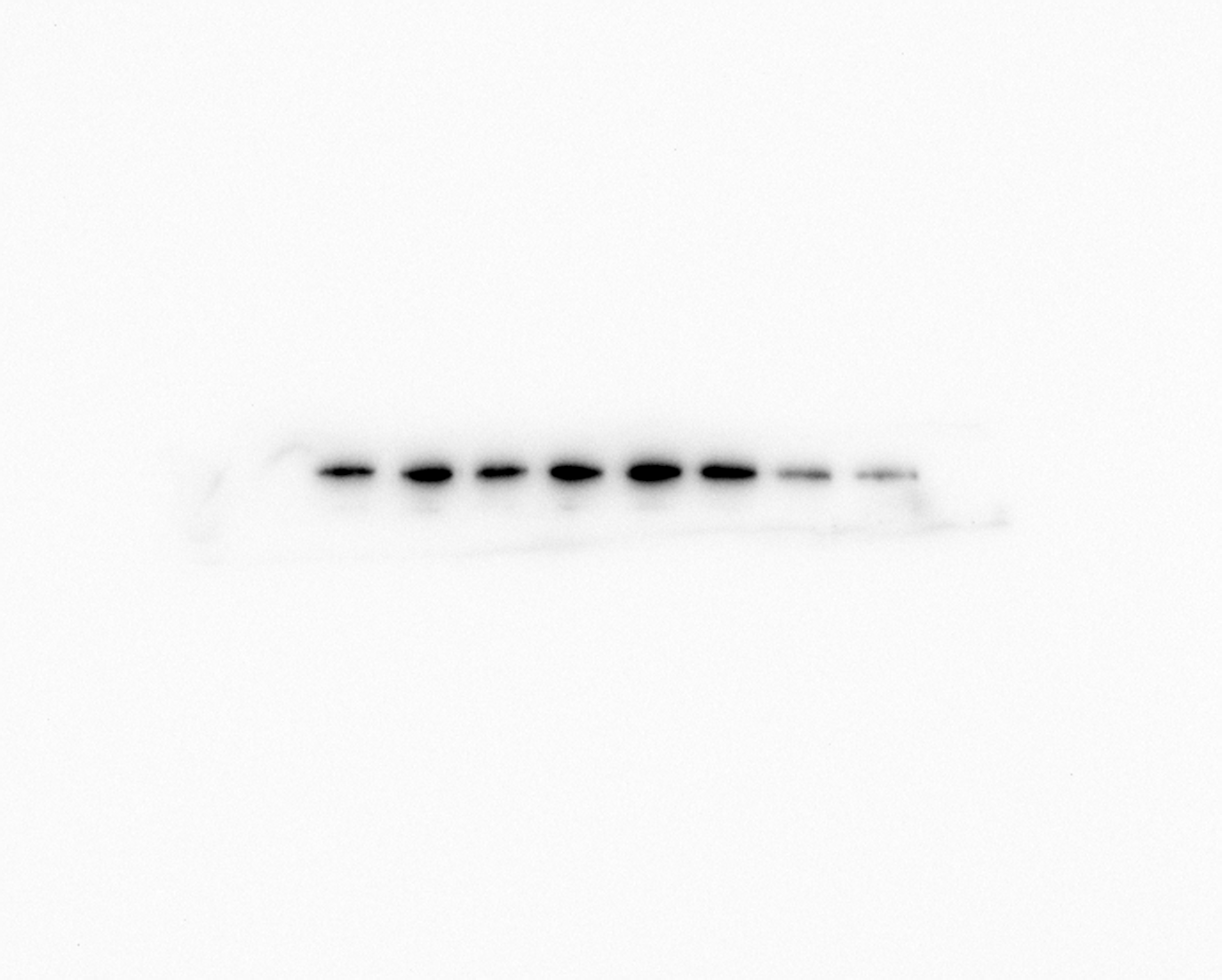
**

**Supplementary FIGURE 2.RIP2**

**
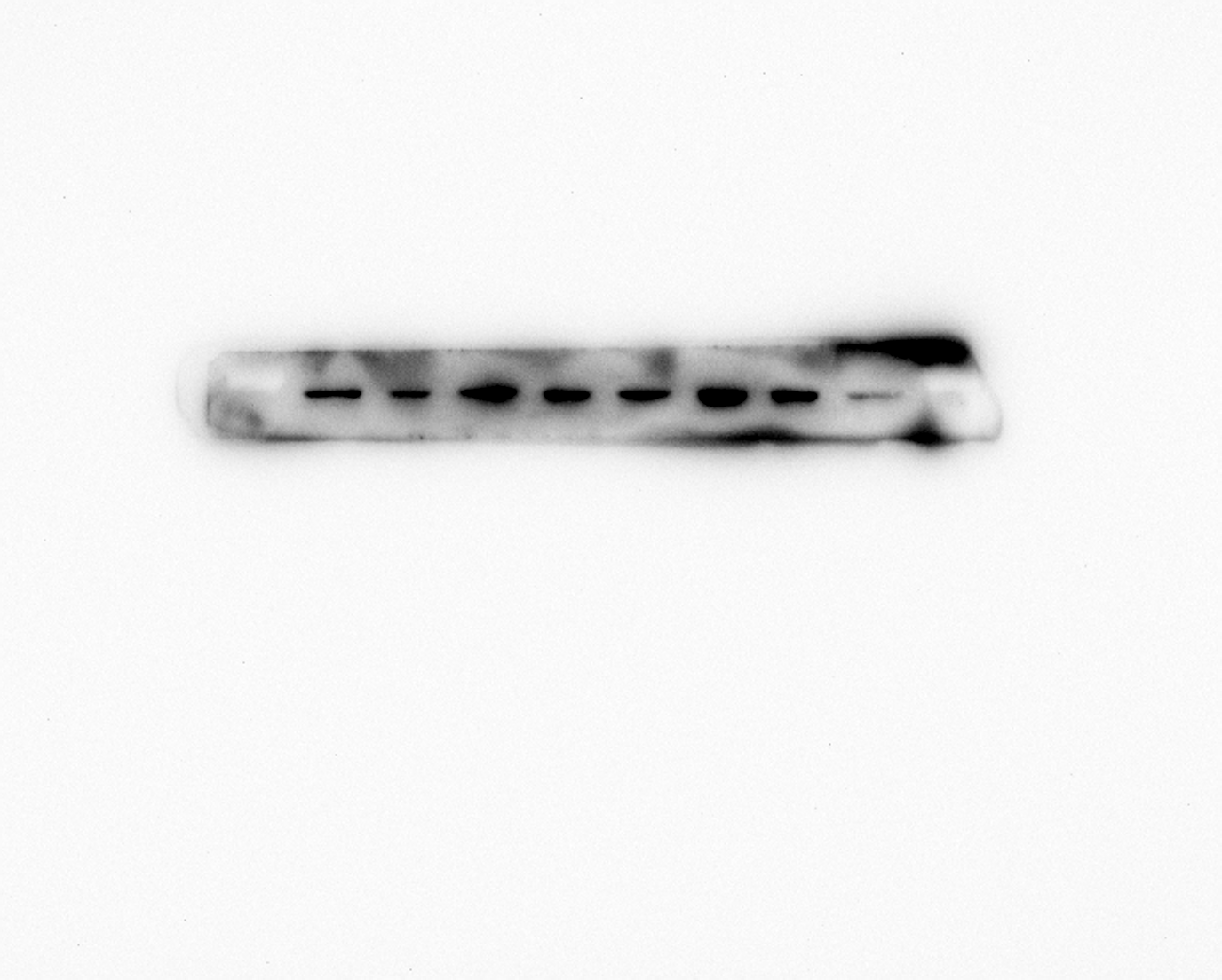
**

**Supplementary FIGURE 3.p-p65**

**
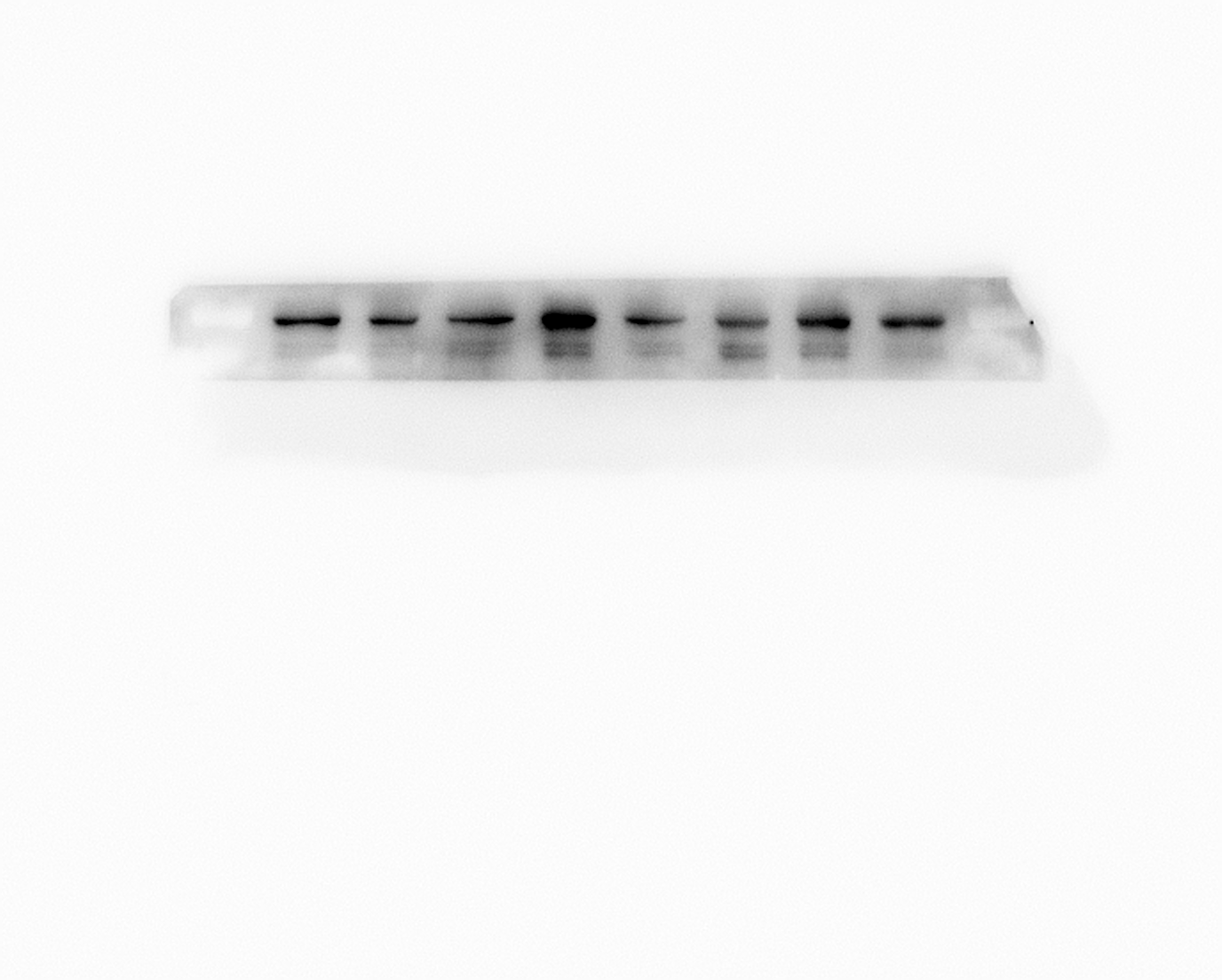
**

**Supplementary FIGURE 4.p65**

**
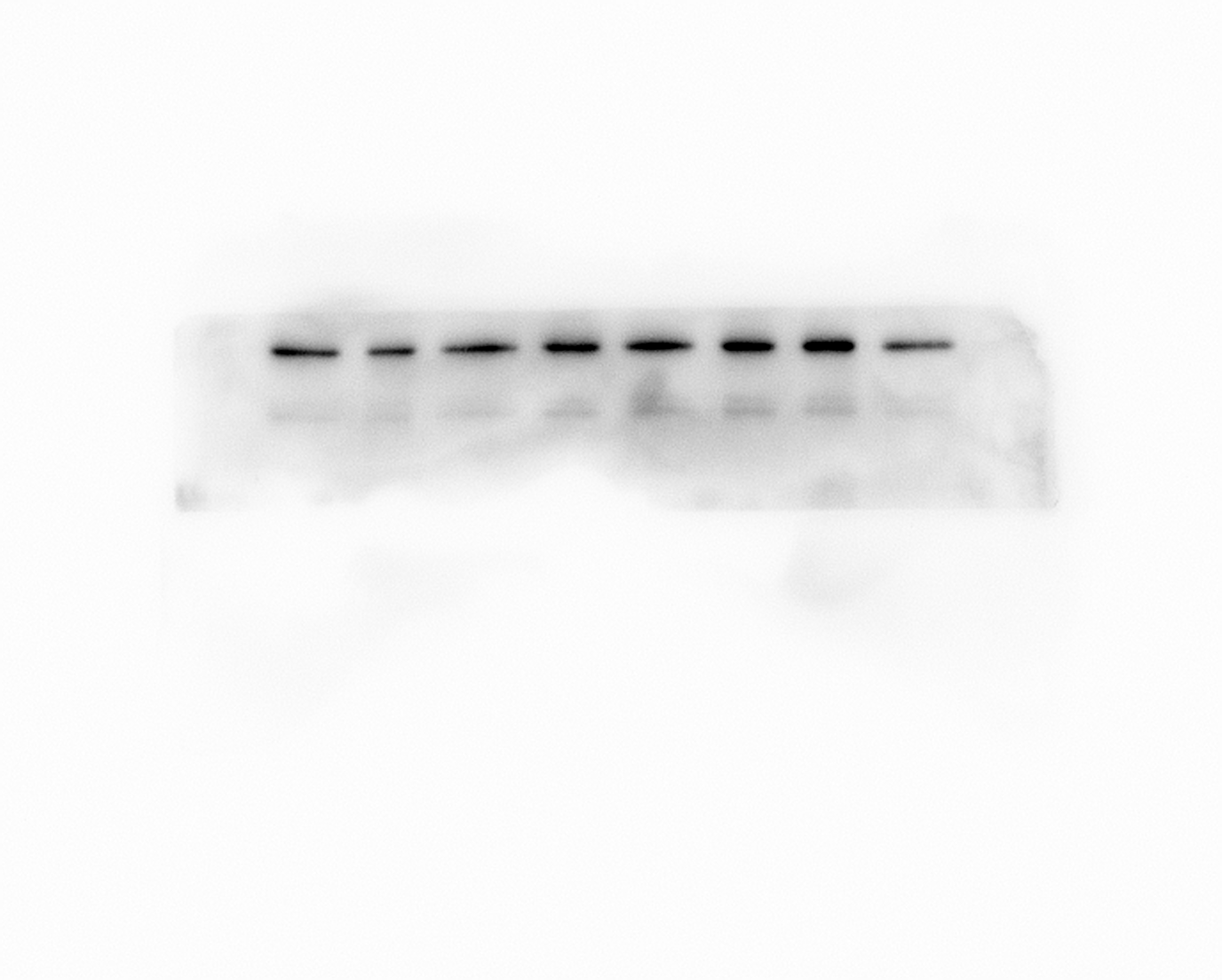
**

**Supplementary FIGURE 5.p-p38**

**
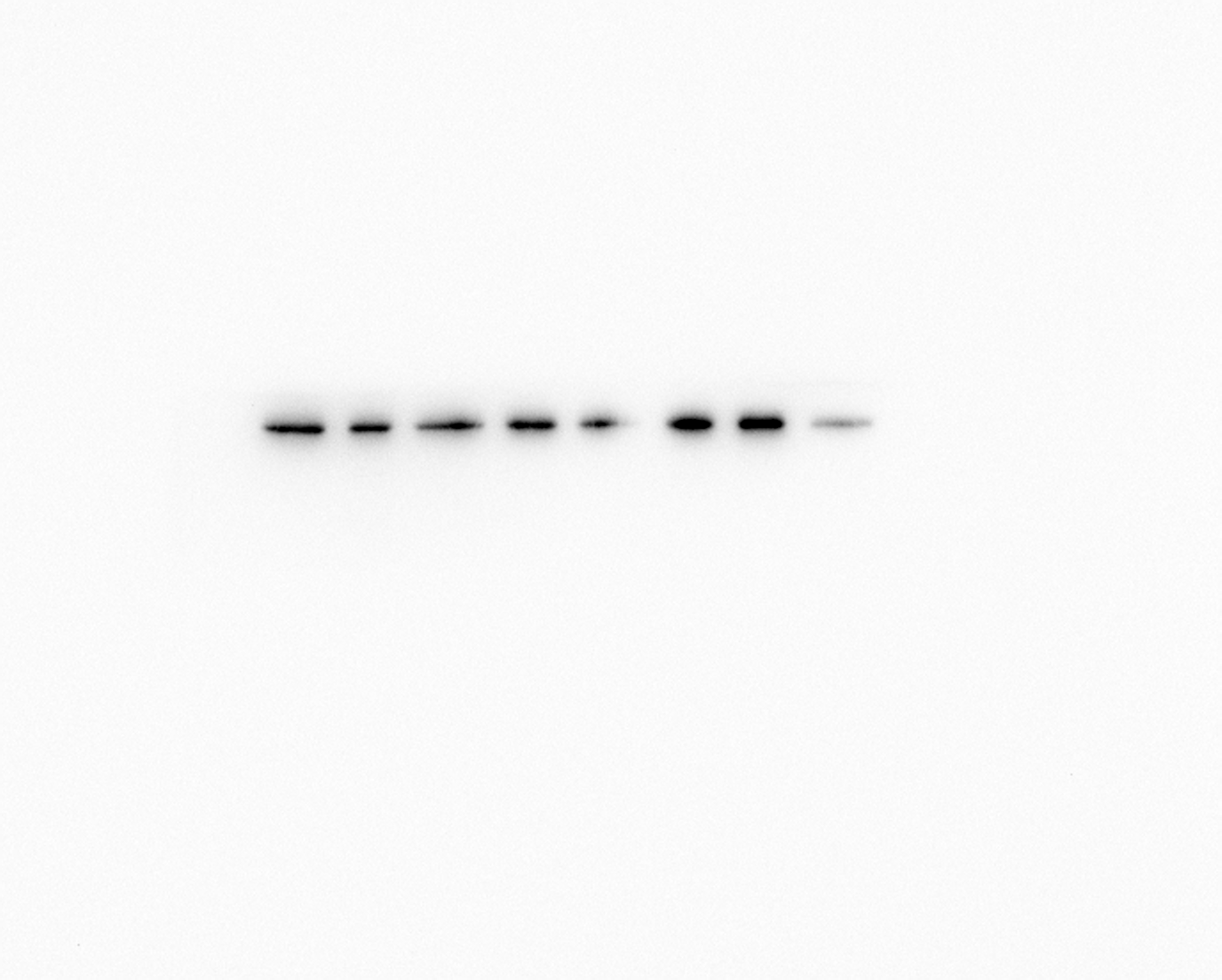
**

**Supplementary FIGURE 6.p38**


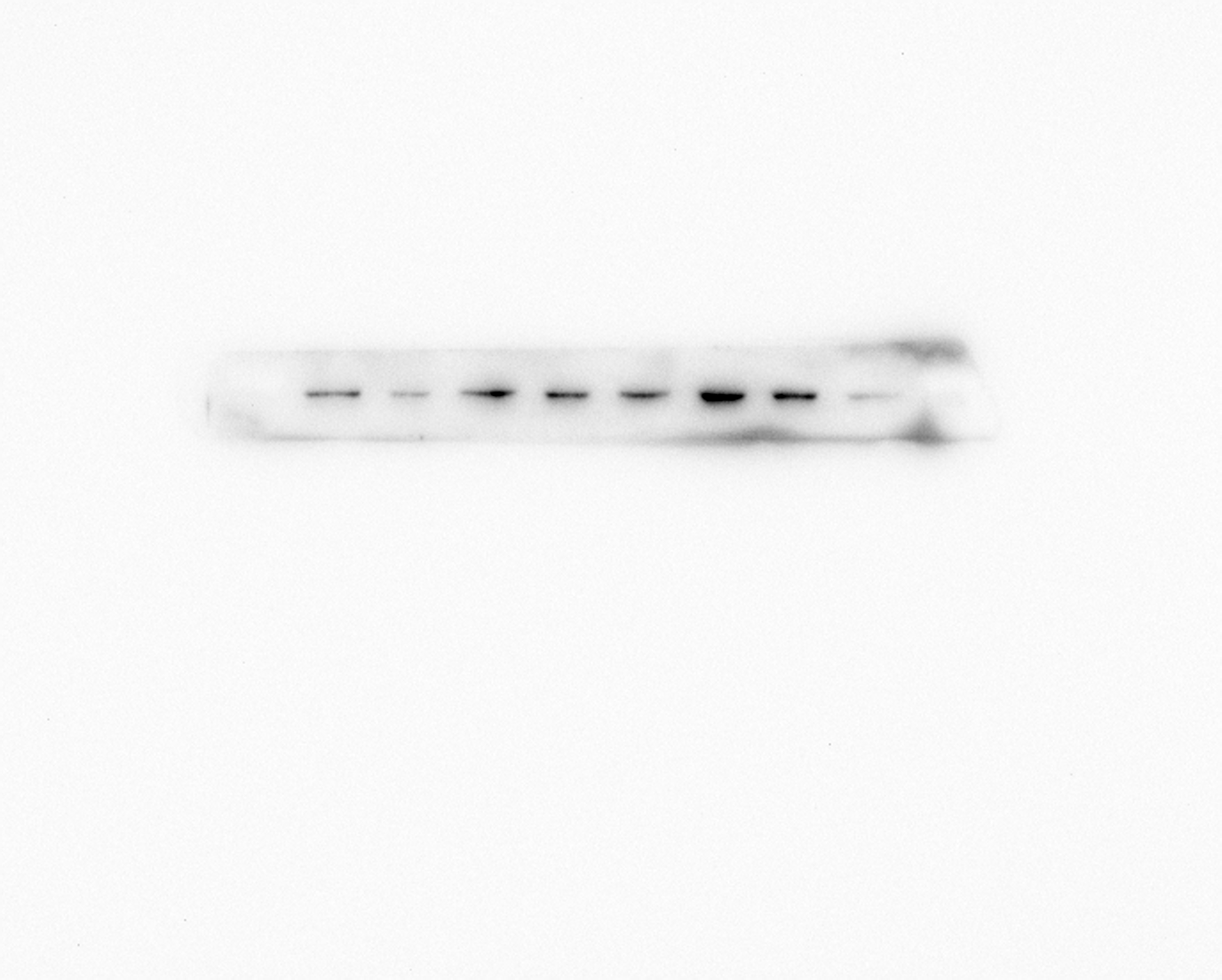


**Supplementary FIGURE 7.p-ERK**

**
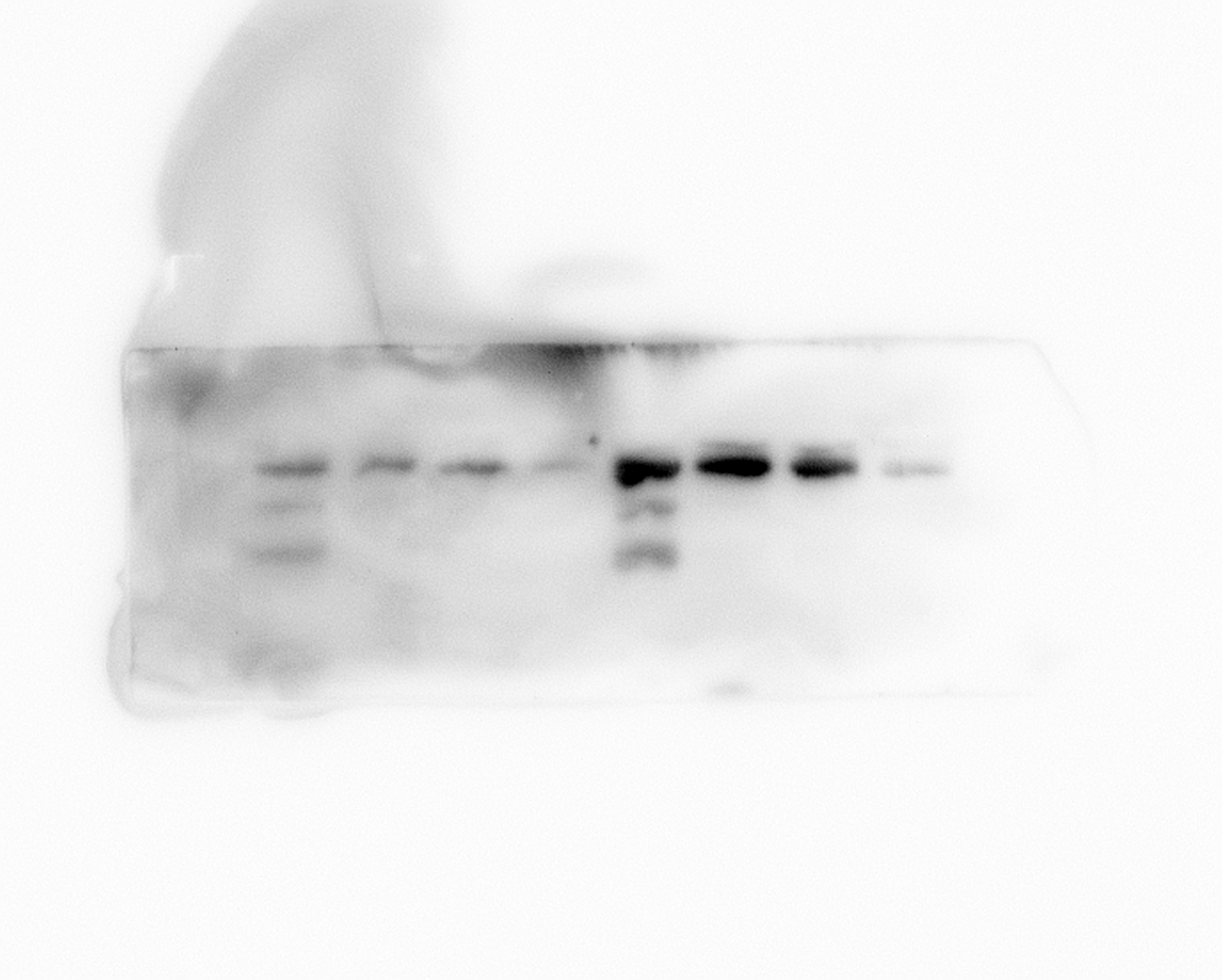
**

**Supplementary FIGURE 8.ERK**


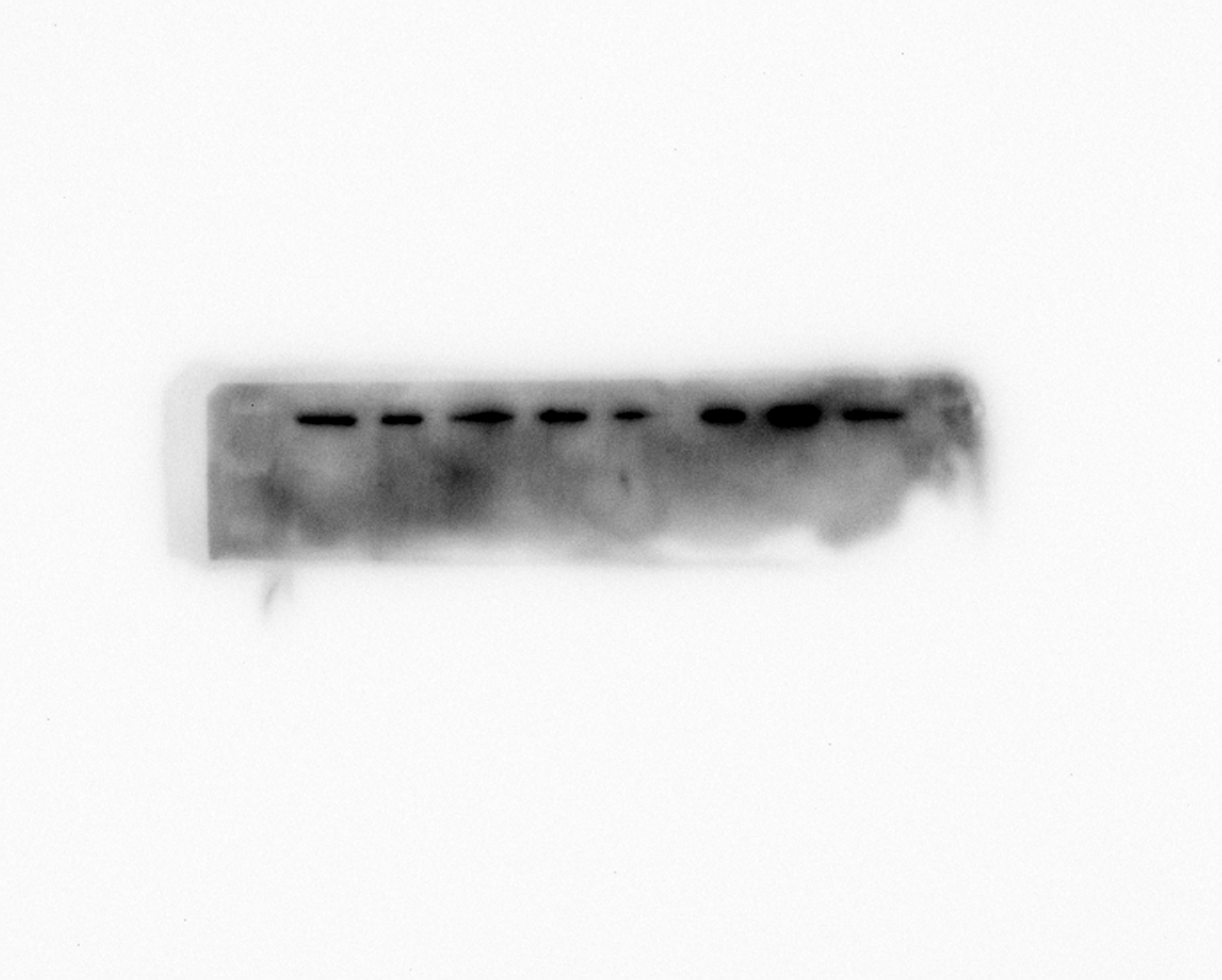


**Supplementary FIGURE 9.p-JNK**

**
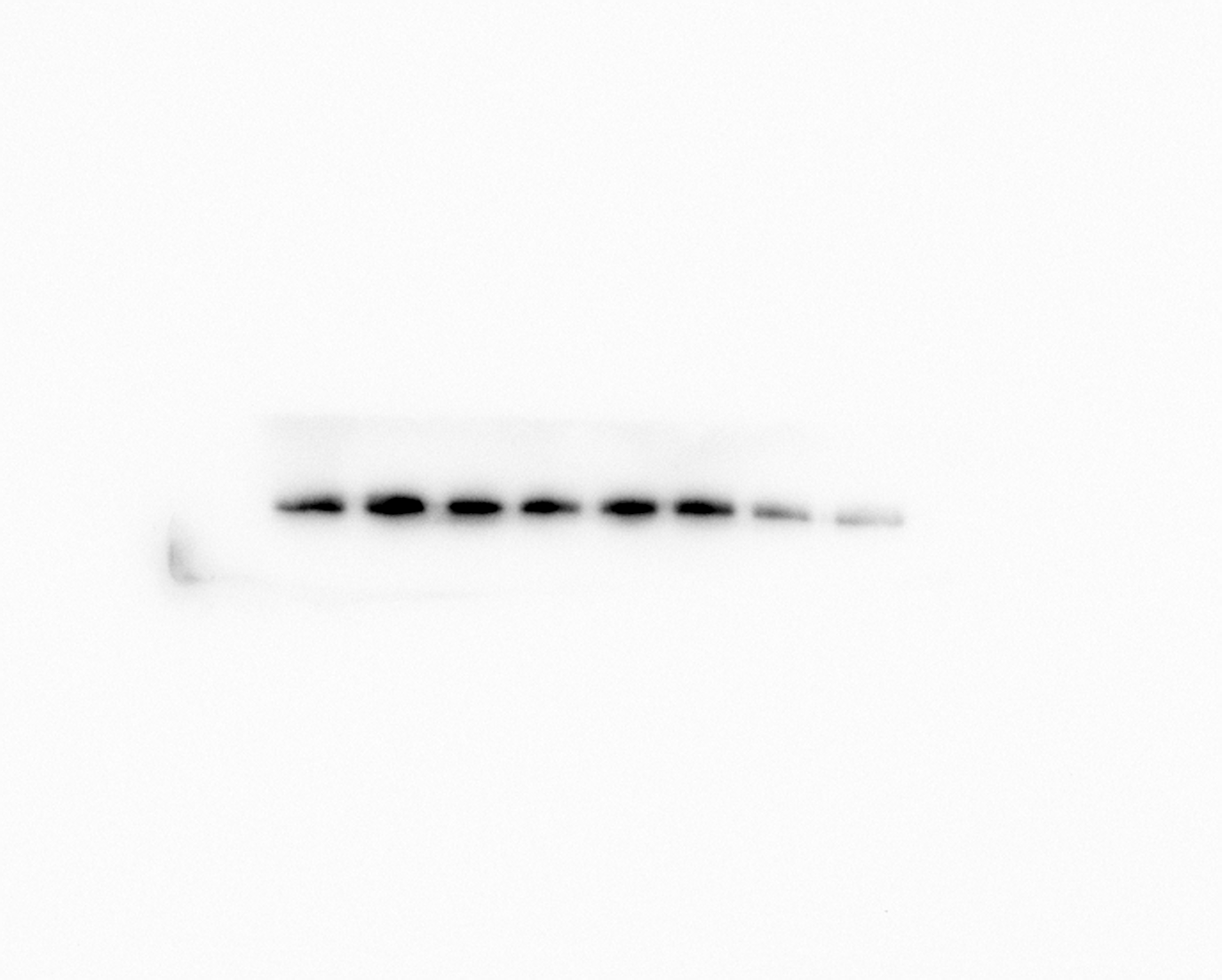
**

**Supplementary FIGURE 10.JNK**

**
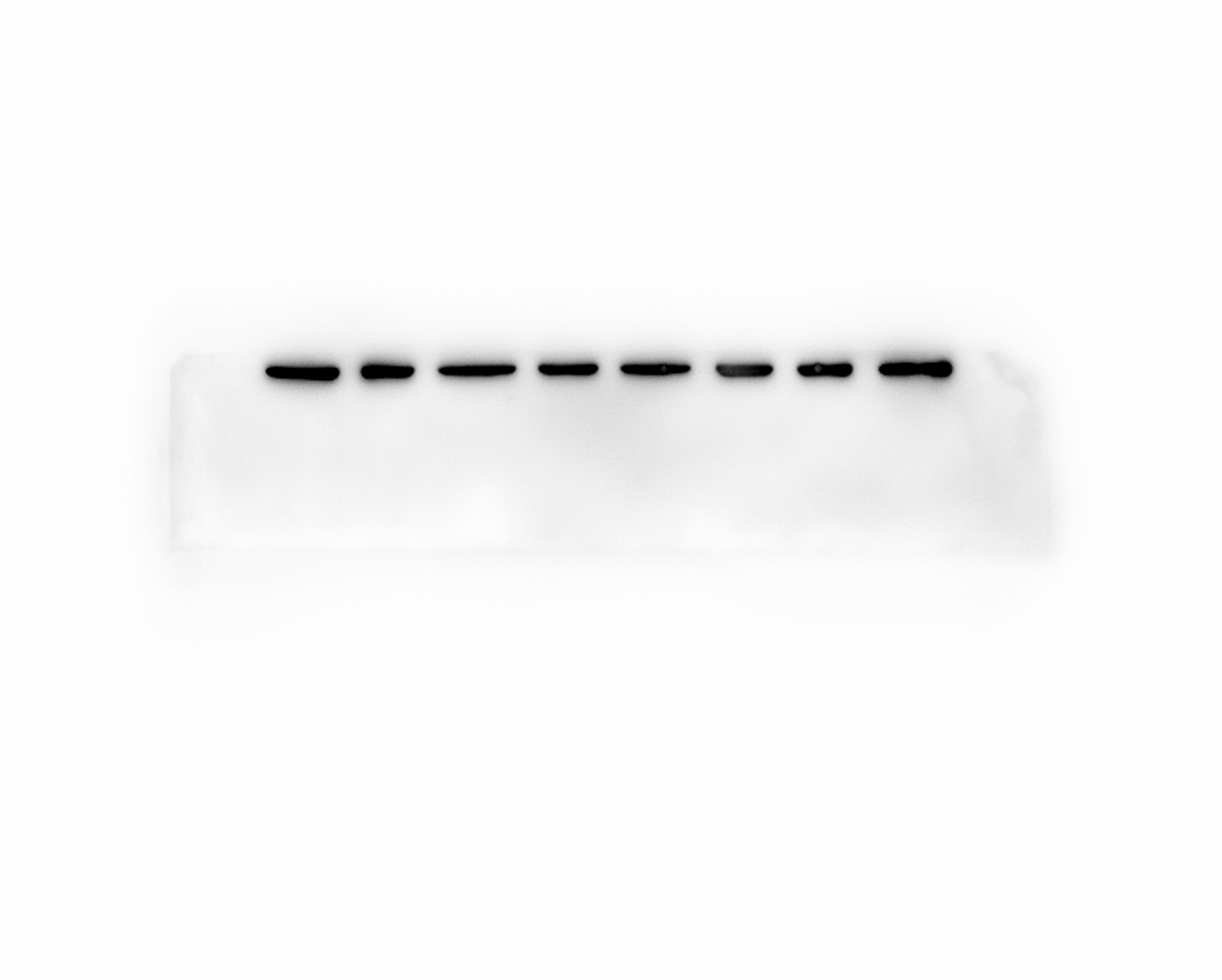
**

**Supplementary FIGURE 11.β-actin**
